# Rift Valley Fever Risk Map Model and Seroprevalence in Selected Wild Ungulates and Camels from Kenya

**DOI:** 10.1371/journal.pone.0066626

**Published:** 2013-06-28

**Authors:** Seth C. Britch, Yatinder S. Binepal, Mark G. Ruder, Henry M. Kariithi, Kenneth J. Linthicum, Assaf Anyamba, Jennifer L. Small, Compton J. Tucker, Leonard O. Ateya, Abuu A. Oriko, Stephen Gacheru, William C. Wilson

**Affiliations:** 1 Center for Medical, Agricultural, and Veterinary Entomology, United States Department of Agriculture, Agricultural Research Service, Gainesville, Florida, United States of America; 2 Biotechnology Center, Kenya Agricultural Research Institute, Nairobi, Kenya; 3 Arthropod-Borne Animal Diseases Research Unit, Center for Grain and Animal Health Research, United States Department of Agriculture, Agricultural Research Service, Manhattan, Kansas, United States of America; 4 National Aeronautics and Space Administration-Goddard Space Flight Center, Greenbelt, Maryland, United States of America; 5 Director of Veterinary Services, Private Bag, Kabete, Kenya; The University of Texas Medical Branch, United States of America

## Abstract

Since the first isolation of Rift Valley fever virus (RVFV) in the 1930s, there have been multiple epizootics and epidemics in animals and humans in sub-Saharan Africa. Prospective climate-based models have recently been developed that flag areas at risk of RVFV transmission in endemic regions based on key environmental indicators that precede Rift Valley fever (RVF) epizootics and epidemics. Although the timing and locations of human case data from the 2006–2007 RVF outbreak in Kenya have been compared to risk zones flagged by the model, seroprevalence of RVF antibodies in wildlife has not yet been analyzed in light of temporal and spatial predictions of RVF activity. Primarily wild ungulate serum samples from periods before, during, and after the 2006–2007 RVF epizootic were analyzed for the presence of RVFV IgM and/or IgG antibody. Results show an increase in RVF seropositivity from samples collected in 2007 (31.8%), compared to antibody prevalence observed from 2000–2006 (3.3%). After the epizootic, average RVF seropositivity diminished to 5% in samples collected from 2008–2009. Overlaying maps of modeled RVF risk assessments with sampling locations indicated positive RVF serology in several species of wild ungulate in or near areas flagged as being at risk for RVF. Our results establish the need to continue and expand sero-surveillance of wildlife species Kenya and elsewhere in the Horn of Africa to further calibrate and improve the RVF risk model, and better understand the dynamics of RVFV transmission.

## Introduction

The Rift Valley fever virus (RVFV) is an arbovirus of the genus *Phlebovirus* of the family Bunyaviridae, and replicates in mosquitoes and in vertebrates [1], [2]. The virus causes Rift Valley fever (RVF), an acute mosquito-borne zoonotic disease affecting animals and humans [3]. The 12 kilobase viral genome consists of a single-stranded, negative-sense tripartite RNA with ambisense polarity [4]–[6]. The L, M, and S segments encode for the RNA-dependent RNA polymerase (RdRp), envelope glycoproteins (Gn/Gc), and nucleocapsid protein (N), respectively [7]. In domestic ruminants, RVF causes high mortality in young animals and sudden onset of abortions in pregnant animals. In humans, uncomplicated RVF cases may present as an acute febrile illness, although more serious complications do occur (ranging from fatal hemorrhagic disease, meningoencephalitis, renal failure, and blindness) [8]–[11] and in some cases death (human case-fatality rate of approximately 0.2 to 5%) [12].

Since the first isolation of the virus in the 1930s there have been multiple epizootics and epidemics in sub-Saharan Africa [13]–[16], in southern and eastern Africa [17], Sahel, West Africa, and in Egypt in 1977 and 1978 [18]. RVFV was thought to be restricted to Africa; however, during 2000 the disease was reported in Yemen and Saudi Arabia [19] but appears not to have become established [20], [21]. RVFV has potential for further international spread due to many factors including climatic changes, human-induced environmental modification (e.g., irrigation, dams, or urbanization), or increases in transportation networks or animal agriculture and trade [22], [23]. The RVF epizootics occur at irregular intervals of 3–15 years, mainly after heavy rains that flood natural depressions in the grasslands of sub-Saharan Africa [24]. The flooding allows hatching of multiple species of *Aedes* mosquitoes, the primary vectors/reservoirs, which eventually feed on nearby vertebrate animals thereby transmitting the virus [25], [26]. The recent RVF epizootic and epidemic in East Africa from 2006–2007 demonstrated that sustained flooding in several parts of the country could have a significant impact on livestock and human health [27]–[29]. In addition, reports have shown that clusters of high RVF seroprevalence encompass areas that experienced previous disease epidemics [30]. The significant role of mosquitoes in RVFV transmission has resulted in the generation of climate-based models to predict the risk of RVF outbreaks within endemic areas in Africa using a combination of temporal and spatial historical records of RVF activity and remotely sensed satellite environmental data, including vegetation indices, sea surface temperatures, and proxy indicators of rainfall, that directly affect RVFV vector mosquito development and survival in RVF-endemic regions [22], [31], [32].

Rift Valley fever infection in humans can be acquired through mosquito bites; however, the primary risk factors are contact with infected domestic animals or animal parts, or consumption of raw meat, blood, or milk [33]–[36]. We have previously shown that human RVF cases are observed in or near zones of elevated risk for RVFV transmission flagged by the climate-based predictive model [31], [32]. This is likely associated with an increased risk of RVFV infection of livestock or wild animals due to development of favorable habitat for mosquito vectors. We have previously demonstrated a relationship between herd management and RVF seroprevalence. Free ranging livestock that may have been removed from or intermittently exposed to mosquito vectors of RVFV were found to have a lower seroprevalence than sedentary livestock herds in high risk areas that may have been subject to persistent exposure to infectious mosquitoes [37]. However, the relationship of RVF seroprevalence in either wild or domestic animals with RVF risk models has not been examined.

There is a need to better understand the potential role of wild mammals in the epidemiology of RVF, especially in regard to the potential inter-epizootic transmission and maintenance of RVFV in enzootic regions [38]. A limited number of studies have examined the prevalence of RVFV antibodies in a range of African wildlife species and similar to this study, most have focused on ungulate species in the orders Artiodactyla and Perissodactyla. For instance, free-ranging black rhinos, African buffalo, and waterbuck in Zimbabwe have been shown to have antibodies to RVFV [39], white rhinos were found to have a high seroprevalence for RVFV antibodies in Kruger National Park, South Africa [18], and RVFV antibodies have been detected in low frequencies in African buffalo sampled from Kenya and South Africa [40], [41]. A serological survey found neutralizing antibodies against RVFV in Kenyan wildlife born during the inter-epidemic period preceding the 2006–2007 outbreak, including African buffalo, black rhino, lesser kudu, impala, African elephant, kongoni (i.e., hartebeest), and waterbuck [42]. However, a much higher seroprevalence was found in wild ruminants, including gerenuk, waterbuck, and eland, in samples collected during the 2006–2007 epizootic and epidemic [42]. Although the potential role of domestic and wild mammals in RVFV maintenance during inter-epidemic periods is not fully understood [27], these studies suggest that surveys of wild animal serology could potentially be leveraged as early indicators of RVFV activity to augment early indicators based on rainfall and vegetation development in models of RVFV transmission risk. Here we describe a retrospective and opportunistic RVF serosurvey of Kenyan wild ungulates from the Order Artiodactyla. The primary objective was to examine temporal and spatial variation in RVF seroprevalence in sampled Kenyan wild ungulates and determine if a preliminary relationship between predicted RVF risk and wildlife seroprevalence could be demonstrated.

## Materials and Methods

### Sera

**Figure 1:**
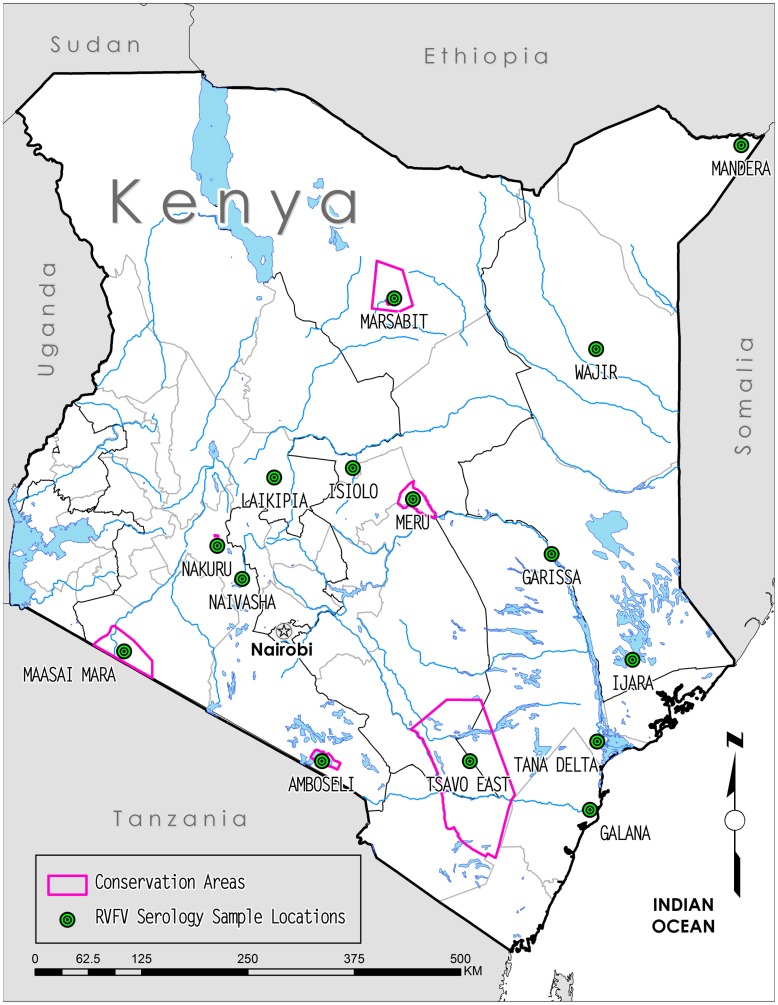
Map of Kenya showing 2000–2009 serology sample locations and relevant Kenya conservation areas.

A total of 840 (wild ungulate (n = 784) and camel (n = 56)) serum samples from 15 Kenya locations (Fig. 1) were obtained from the Veterinary Laboratories in Kabete, Kenya. These samples were collected between 2000 and 2009 for disease surveillance (especially PPR and Rinderpest) not directly related to this project, but provide an unbiased sample collection with regard to temporal and spatial continuity. These serum samples span the pre-epizootic, epizootic, and post-epizootic periods and regions of the 2006–2007 RVF outbreak in Kenya and are a unique resource to preliminarily examine possible alignment of RVF risk predictions and observed RVF activity. Of these, 183 samples had been collected over a period of seven years (2000–2006) preceding the outbreak from 6 locations, 299 samples had been collected in 2007 during the outbreak from 5 locations, and 358 samples had been collected in 2008–2009 after the outbreak from 8 locations (Table 1). The serum samples were coded (location, species, date, and animal number) and stored in aliquots at −80°C. The age and health status of the sampled animals are not known. Precise geolocations of samples were not recorded during sampling; therefore locations were estimated using location names associated with each sample. Seven of the 15 locations, Lake Naivasha, Marsabit, Meru, Lake Nakuru, Maasai, Tsavo East, and Amboseli are well-known conservation areas in Kenya and geolocation for samples collected from these areas was estimated as centroid or the urban center of the conservation area boundary polygon (Fig. 1). For the remaining 8 locations, Mandera, Wajir, Laikipia, Isiolo, Garissa, Ijara, Tana Delta, and Galana were taken as the point location of the urban centers for the samples with these names (Fig. 1). In cases where samples had similar, but not identical location names, for example Marsabit and Marsabit Lodge (Table 1) or Nakuru and Lake Nakuru (Table 1), these samples were grouped into a single location, for example “Marsabit” or “Nakuru”, for mapping purposes (Fig. 1).

**Table 1:** Wildlife sera collected in different locations during pre-epizootic, epizootic, and post epizootic RVF outbreak in Kenya.

Sampling period	Area	Species	Date of collection	Total samples collected	Positive samples	% Positive samples
2000–2006 (pre-epizootic) period	Tsavo	buffalo	11/10/2000	10	0	0
	Maasai Mara	buffalo	14/10/2000	2	0	0
	Galana	**camel**	14/10/2000	15	**1**	**6.7**
	Garissa	**camel**	10/11/2000	13	**1**	**7.7**
	Laikipia	buffalo	28/7/2004	49	0	0
	Amboseli National Park	eland	26/11/2006	15	0	0
		giraffe	26/11/2006	16	0	0
		**waterbuck**	26/11/2006	17	**2**	**11.8**
		**buffalo**	26/11/2006	21	**1**	**4.8**
		kongoni	26/11/2006	14	0	0
		**gazelle**	26/11/2006	11	**1**	**9.1**
	**Total**			**183**	**6**	**3.3%**
2007 epizootic period	Lake Naivasha National Park	**buffalo**	6/1/2007	27	**11**	**40.7**
	Laikipia	waterbuck	2/1/2007	2	0	0
		eland	23/1/2007	3	0	0
		giraffe	23/1/2007	8	0	0
		**buffalo**	23/1/2007	111	**9**	**8.1**
	Nakuru	**waterbuck**	2/2/2007	4	**1**	**25**
		warthog	2/2/2007	1	0	0
	Lake Nakuru	gazelle	2/2/2007	26	0	0
	Maasai Mara	waterbuck	2/2/2007	1	0	0
		gazelle	2/2/2007	1	0	0
		**buffalo**	2/2/2007	25	**15**	**60**
		**eland**	2/2/2007	1	**1**	**100**
		**giraffe**	2/2/2007	7	**2**	**28.6**
		**gerenuk**	2/2/2007	6	**4**	**66.7**
		**warthog**	2/2/2007	42	**32**	**76.1**
	Lake Naivasha National Park	**impala**	2/6/2007	2	**2**	**100**
		**waterbuck**	3/6/2007	4	**2**	**50**
	Isiolo Webera	**camel**	28/6/2007	28	**16**	**57.1**
	**Total**			**299**	**95**	**31.8%**
2008–2009 (post-epizootic) period	Wajir	**warthog**	18/2/2008	11	**1**	**9.1**
	Marsabit	warthog	18/2/2008	2	0	0
	Mandera	warthog	18/2/2008	12	0	0
	Wajir	giraffe	8/9/2008	10	0	0
	Marsabit Lodge	giraffe	8/9/2008	2	0	0
	Mandera East	giraffe	8/9/2008	2	0	0
	Wajir	warthog	8/9/2008	8	0	0
	Ijara (Kotile)	warthog	8/11/2008	7	0	0
	SBT Klegdela	**warthog**	11/11/2008	22	**1**	**4.6**
	Marsabit	buffalo	11/11/2008	2	0	0
	Meru Conservation Area	**buffalo**	11/11/2008	61	**1**	**1.6**
	Tana Delta	**buffalo**	11/11/2008	40	**2**	**5**
	Meru Conservation Area	**waterbuck**	11/11/2008	6	**1**	**16.7**
	Tsavo East	giraffe	27/11/2008	5	0	0
		lesser kudu	27/11/2008	2	0	0
	Meru Conservation Area	eland	27/11/2008	3	0	0
	Meru Conservation Area	**warthog**	27/11/2008	4	**1**	**25**
	Ijara (Hare)	warthog	27/11/2008	30	0	0
	Tsavo East	**buffalo**	27/11/2008	89	**9**	**10.1**
		**waterbuck**	27/11/2008	7	**1**	**14.3**
	Wajir	gerenuk	17/2/2009	2	0	0
	Meru Conservation Area	**giraffe**	18/2/2009	29	**1**	**3.5**
	Ijara (Hulugho)	gerenuk	18/2/2009	2	0	0
	**Total**			**358**	**18**	**5%**

The results of the analysis of the RVFV antibodies in the samples by recombinant N inhibition ELISA are indicated.

The samples used in this study were diagnostic specimens sent to the Kenya Department of Veterinary Services, Veterinary Laboratory in Kabete, Kenya. These samples were taken by the Kenyan Wildlife Services staff according to their national standards. The authors conducted no animal handling or sampling. Samples were used with permission from the Kenyan Department of Veterinary Services and Wildlife Services.

### Inhibition ELISA Test

The procedure for inhibition ELISA that detects both IgG and IgM used in this study was based on a previously described method [43]. Briefly, polystyrene ELISA microtitre plates (MaxiSorp, Nunc™, Denmark) were coated with 100 µl per well with polyclonal sheep anti-RVFV capture antibody in 0.01 M phosphate buffered saline (PBS), pH 7.4, overnight in a humidity chamber at 4°C. The plates were washed four times with PBS/0.05% Tween-20; the same washing step was performed for all subsequent washing steps. Plates were blocked with 10% non-fat milk/PBS (1 h; 37°C). During the blocking stage, undiluted test and control sera were each added into diluting wells containing virus or control antigen pre-diluted in 2% skim milk in PBS. Test and control sera/virus antigen mixture was added to rows A–D 1–12 and test and control sera/control antigen mixture to rows E–H 1–12 and incubated for 1 h in a moist chamber at 37°C. The plates were then washed, followed incubation with mouse anti-RVFV antibodies (1 h; 37°C). After washing, the plates were incubated with anti-mouse IgG HRPO-conjugate diluted 1∶2000 (1 h; 37°C). The plates were washed six times as before, and developed by addition of 2,2′-azino-bis(3-ethylbenthiazoline-6-sulphonic acid) (ABTS) substrate. Optical densities (O.D) were then measured in a Multiskan EX^R^ plate reader (Thermo Electron Corp.) using a 405 nm filter.

### Serology Scoring

The specific activity of each serum (net optical density, O.D.) was calculated by subtracting the non-specific background OD in the wells with control antigen from the specific O.D. in wells with virus antigen. The mean OD readings for replicate tests were converted to a percentage inhibition (PI) value. Sera that gave more than 34.2% inhibition (O.D. neg.−4.26×s.d., *P* = 0.001; where s.d. = 0.05) were scored as seropositive for RVFV antibodies.

### RVF Risk Maps

The methods for producing RVF risk maps have been described in detail elsewhere [22], [31], [32], [44] and are only summarized here. Prospective risk analysis for RVF activity is triggered by changes in global climate patterns indicated by sea surface temperatures and the El Niño/Southern Oscillation phenomenon that predict high likelihood of prolonged and above-normal rainfall in RVF-endemic regions of Africa and the Arabian Peninsula within 2 to 5 months [17], [22], [31]–[33]. In most RVF-endemic regions, excess and prolonged rainfall has a positive linear relationship with vegetation development [45]–[47]. Photosynthetic activity resulting from vegetation development produces characteristic reflections of solar radiation that are routinely recorded by satellite instruments and used to calculate the normalized difference vegetation index (NDVI) [48], [49]. The “greening” of habitat indicated by increases in NDVI in turn has a direct relationship with RVFV mosquito vector development, emergence, and survival [45]. Specifically, 3 months of sustained excess rainfall and resultant persistent increases in NDVI are strongly indicative of impending high levels of RVFV transmission in large part due to extremely favorable vector mosquito habitat [16], [45].

The boundaries of the risk maps are set by creating a spatial mask that defines the potential epizootic area (PEAM) [16], [31], [32], [44]. This mask is derived by a thresholding method on NDVI climatological values that range between 0.15–0.4 NDVI units. A map derived from the thresholding method identifies the savanna complexes of Africa, which are subject to extremes in interannual climate variability. The mask includes those areas that receive between 200–800 mm/yr of rainfall. These are primarily the regions where RVF outbreaks have been described, especially in East Africa, Southern Africa, and the Sahel region [50]. Thus, the spatial boundaries of the model are set to include mainly areas that possess the environmental infrastructure for RVFV vector development and host domestic animals. The PEAM is shown in green color on RVF risk map figures and designated the “Potential Epizootic Region.” Areas outside the PEAM include areas that may not possess obvious appropriate habitat but some may have nevertheless experienced RVFV transmission. Within the regions highlighted by the PEAM, the risk model flags 1 km^2^ blocks that show 3 month persistence in above-normal NDVI data from SPOT-Vegetation optical instruments on board SPOT-4 and -5 satellites as RVF risk areas. Above-normal NDVI is derived by comparing the percentage departure of current month NDVI values against 10-year mean NDVI values for that month in a 3-month moving window. Maps are output from the model on a monthly basis showing the RVF potential epizootic area and flagged RVF risk areas. As patterns of rainfall and vegetation development unfold over time, areas at risk of RVF may emerge or fade with these changing conditions as the monthly risk estimates are produced.

To match the temporal framework of the serological samples we extracted mapped RVF risk assessments for the month samples were collected as well as the preceding month, given that antibodies detected in a month could have been the result of exposure in a prior month. Estimated serology sample locations for each sample month were then mapped with the RVF risk assessments, and we examined each sample month and prior month for spatial co-occurrence of seropositivity and predicted RVF risk.

## Results

**Figure 2:**
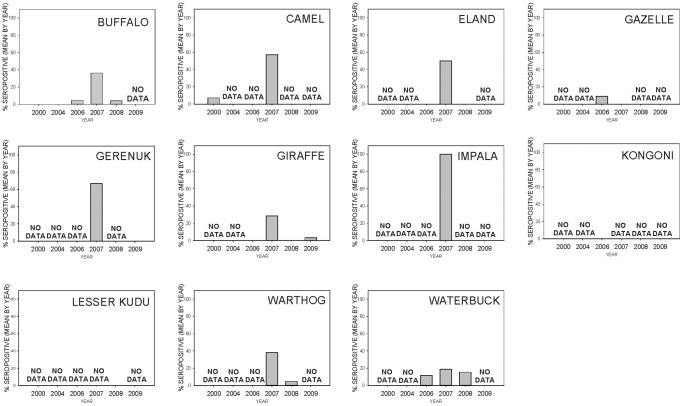
Bar graphs showing by-species patterns of change in RVFV seropositivity before, during, and after the 2006–2007 RVF epizootic.

**Figure 3:**
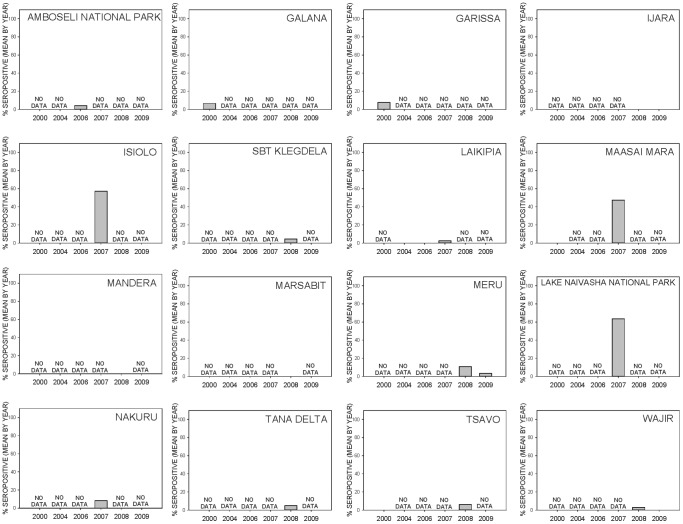
Bar graphs showing by-location patterns of change in RVFV seropositivity before, during, and after the 2006–2007 RVF epizootic.

**Figure 4:**
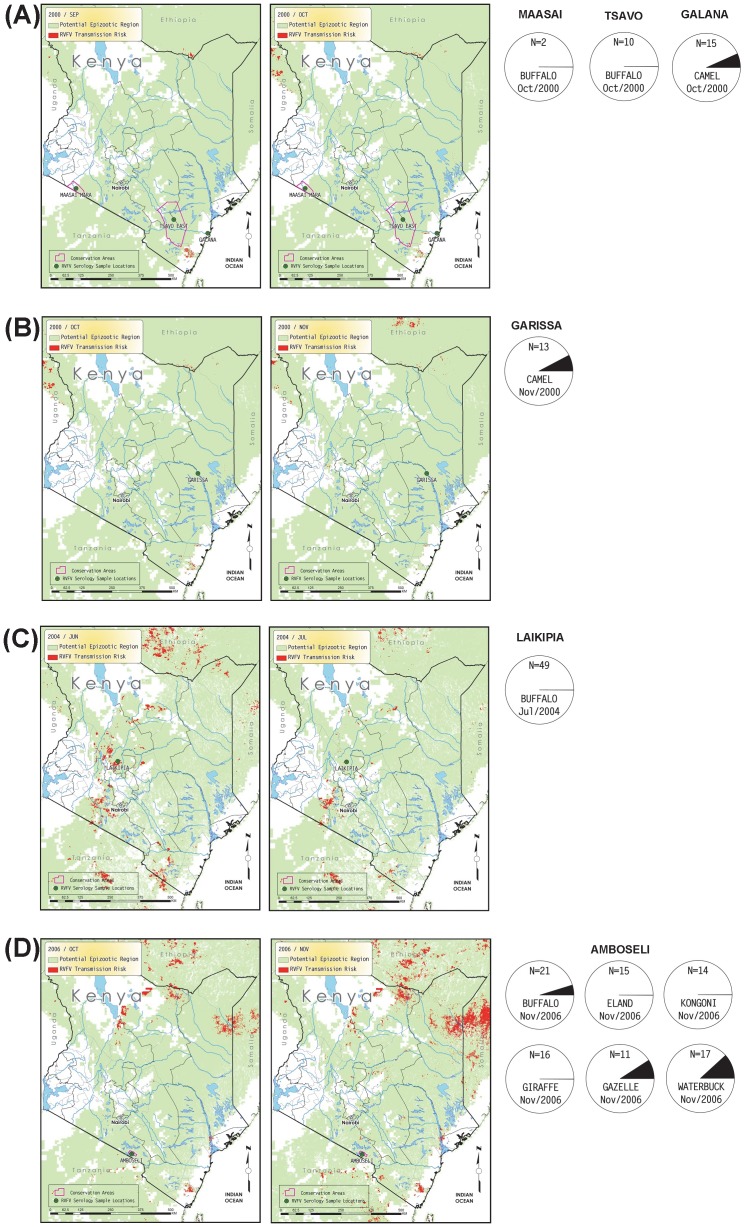
Monthly predicted RVF risk assessment map overlaid with serological results collected prior to the 2006–2007 RVF outbreak; A) Tsavo East in September and October 2000, B) Garissa in October and November 2000, C) Laikipia in June and July 2000, and D) Amboseli in October and November 2000. The light green background color shows the extent of the potential epizootic region and high risk is indicated by red color in 1 km^2^ pixels. Magenta lines represent polygons for conservation areas such as national parks or preserves. For each sample month, the left-hand map shows the RVF risk conditions for the prior month, and the right-hand map shows the month the samples were taken, along with sample locations. Below the maps for sample months, pie charts show the proportion of samples found to be RVF seropositive for each species, by location. Only locations sampled in that month are plotted.

**Figure 5:**
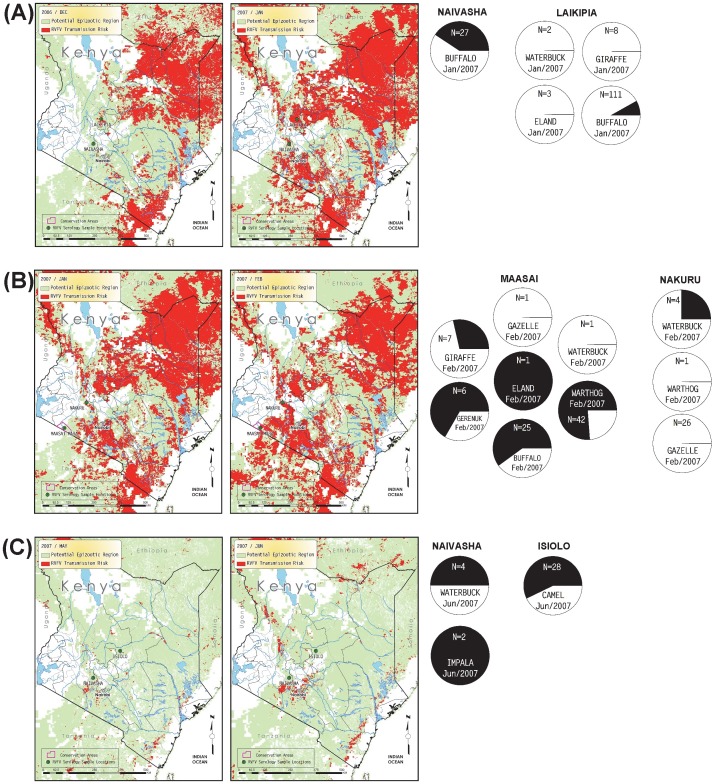
Monthly predicted RVF risk assessment map overlaid with serological results collected during the 2006–2007 RVF outbreak; A) Laikipia and Naivasha in December and January 2006, B) Maasai and Nakuru in January and February 2007, and C) Isiolo and Naivasha in May and Jun 2007. See Fig. 4 for descriptive legend.

**Figure 6:**
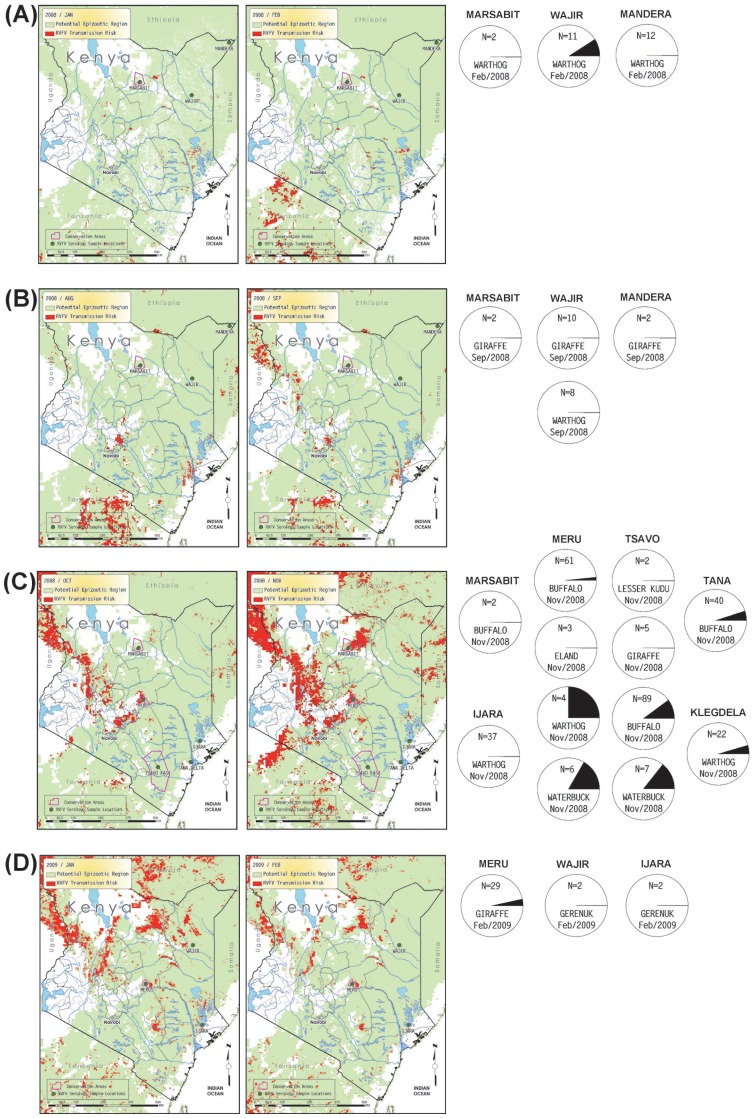
Monthly predicted RVF risk assessment map overlaid with serological results collected after the 2006–2007 RVF outbreak; A) Marsabit, Mandera, and Wajir in January and February 2008, B) Marsabit, Mandera, and Wajir in August and September 2008, C) Ijara, Klegdela, Marsabit, Meru, Tana, and Tsavo in October and November 2008. See Fig. 4 for descriptive legend.

The 840 serum samples analyzed in this study were collected from four families within the order Artiodactyla. Wild ungulate species tested include African buffalo (*Syncerus caffer*, n = 437), warthog (*Phacochoerus africanus*, n = 139), waterbuck (*Kobus ellipsiprymus*, n = 41), giraffe (*Giraffa camelopardalis*, n = 79), gazelle (*Gazella* sp., n = 38), common eland (*Tragelaphus oryx*, n = 22), kongoni or hartebeest (*Alcelaphus buselaphus*, n = 14), gerenuk (*Litocranius walleri*, n = 10), impala (*Aepyceros melampus*, n = 2), and lesser kudu (*Tragelaphus imberbis*, n = 2). Although a domesticated species, samples from dromedary camels (*Camelus dromedarius*, n = 56) were also included in this study. The sampling sites consisted of 7 conservation areas, such as national parks and game reserves, and 8 rural regions in Kenya (Fig. 1). Year-to-year patterns of change in percent seropositive samples by species and by locations are shown in Figs. 2 and 3, respectively. Specific seropositive percentages by sample site per species by sample month and year are shown as pie charts with relevant maps of RVF risk assessments (Figs. 4–6).

The total number of wildlife serum samples analyzed for presence of RVFV antibodies during the pre-epizootic (2000–2006), epizootic (2007), and post-epizootic (2008–2009) periods are indicated in Table 1. Although the RVF outbreak studied here is recorded as a 2006–2007 outbreak, the samples taken in November, 2006, are grouped as pre-epizootic samples because (a) the November, 2006, rate of seropositivity more closely matches rates in the earlier 2000–2006 pre-epizootic samples, (b) the RVF risk assessments for October and November, 2006, were low in southern Kenya and more indicative of pre-epizootic conditions and (c) the outbreak began in December 2006.

Approximately 5% (n = 40) of the samples collected in 2000 and 4.3% (n = 94) of the 2006 samples were found to be positive for RVFV antibodies by the inhibition ELISA test, while 0% (n = 49) of the samples collected in 2004 were positive (Table 1). Serologically positive samples in the 2000–2006 pre-epizootic period were detected in camel (7.2%, n = 28), waterbuck (11.8%, n = 17), buffalo (1.2%, n = 82), and gazelle (9.1%, n = 11). It is noteworthy that none of the giraffe (n = 16), common eland (n = 15), or kongoni (n = 14) samples were seropositive for RVFV during the pre-epizootic period (Table 1, Figs. 2–3).

In contrast to pre-epizootic samples, 31.8% (n = 299) of the 2007 samples taken during the RVF epizootic were found to be positive. For the 2007 epizootic period, serologically positive samples were detected in camel (57.1%, n = 28), waterbuck (27.3%, n = 11), impala (100%, n = 2), buffalo (21.5%, n = 163), common eland (25%, n = 4), giraffe (13.3%, n = 15), gerenuk (66.7%, n = 6), and warthog (74.4%, n = 43). Interestingly, 2 of 2 (100%) samples collected from impala at Lake Naivasha National Park were positive and the one (100%) common eland sample collected from Maasai Mara National Park was also positive, although these results should be interpreted with caution due to very small sample sizes. Higher percentages of RVFV seropositivity were detected from gerenuk (66.7%, n = 6), warthog (76.2%, n = 42), buffalo (60%, n = 25), and giraffe (28.6%, n = 7) sampled specifically from Maasai Mara National Park. The RVF seroprevalence during the epizootic were from domestic camel (57.1%, n = 28) sampled from Isiolo Webera and waterbuck (50%, n = 4) and buffalo (40.7%, n = 27) sampled from Lake Naivasha National Park. No RVFV antibody was detected in gazelle during the epizootic period (Table 1, Figs. 2–3).

From the samples of wildlife sera analyzed from the 2008–2009 post-epizootic period, there was a reduction in RVFV seropositive samples detected by inhibition ELISA. Of the 325 serum samples analyzed from 2008, only 5.2% were positive for RVFV antibodies, and of the 33 samples analyzed from 2009, only 3% were seropositive. The majority of seropositive samples detected in 2008–2009 were from giraffe (2.1%, n = 48), warthog (3.1%, n = 96), buffalo (6.3%, n = 192), and waterbuck (15.4%, n = 13). The highest single-location RVF seroprevalence result for the 2008–2009 post-epizootic period were detected in warthog (25%, n = 3) and waterbuck (16.7%, n = 6) from the Meru Conservation Area, and waterbuck (14.3%, n = 7) from Tsavo East National Park. The remaining samples from 2008–2009 were generally less than 10% seropositive. No positive sample was detected from samples collected from lesser kudu (n = 2), gerenuk (n = 4), or common eland (n = 3) in the post-epizootic period (Table 1, Figs. 2–3).

Relevant monthly outputs from the RVF risk assessment model are mapped in Fig. 4 (pre-epizootic period), Fig. 5 (epizootic period), and Fig. 6 (post-epizootic period). The key observations across Figs. 4–6 are whether locations with serology values >0 for any sampled ungulate species co-occur with map pixels flagged for risk of RVFV transmission. Although pixels flagged for RVFV transmission risk may not be at or directly adjacent to plotted sample locations, we may consider risk-flagged pixels within an arbitrary radius of 75 km from each location – not only because all plotted locations are only estimates of the actual sample locations, which were not recorded with the serosamples, but also because the animals range freely and may not have become exposed to RVFV where they were sampled. In some months shown in Figs. 4–6, the density of risk pixels is very low across the landscape, and in other months the density is high. For each location we ran a GIS analysis that counted the number of flagged pixels in a 75 km radius and the area at risk for each sample month. Results shown in Table 2 support the direct observation that in the pre-epizootic and post-epizootic periods, the density of risk pixels was lower, and the frequencies of seropositive samples were lower, than during the epizootic period.

**Table 2:** RVF risk quantified in a 75 km radius from each sample location.

					AREA OF RVF RISK (km^2^ in 75 km radius)		% AREA OF RVF RISK (km^2^ risk/17,671 km^2^)	
Period	Year	Month	Area	% Seropositive	(month before sample)	(month of sample)	(month before sample)	(month of sample)
**Pre-**	2000	OCT	Galana	6.7	13	***85***	0.1	**0.5**
**Epizootic**			Maasai	0	0	**0**	0	**0**
			Tsavo	0	0	**2**	0	**0**
		NOV	Garissa	7.7	2	**2**	0	**0**
	2004	JUL	Laikipia	0	1,324	**58**	7.5	**0.3**
	2006	NOV	Amboseli	4.3	157	**285**	0.9	**1.6**
		*Totals, Pre-Epizootic:*		*3.3%*	*1,496*	***432***	***–***	***–***
**Epizootic**	2007	JAN	Naivasha	40.7	173	**2,017**	1.0	**11.4**
			Laikipia	7.3	1,067	**7,941**	6.0	**44.9**
		FEB	Maasai	65.1	510	**4,020**	2.9	**22.7**
			Nakuru	3.2	1,501	**4,216**	8.5	**23.9**
		JUN	Naivasha	66.7	842	**1,790**	4.8	**10.1**
			Isiolo	57.1	87	**167**	0.5	**0.9**
		*Totals, Epizootic:*		*31.8%*	*4,180*	***20,151***	***–***	***–***
**Post-**	2008	FEB	Marsabit	0	146	**85**	0.8	**0.5**
**Epizootic**			Wajir	9.1	22	**0**	0.1	**0**
			Mandera	0	1	**0**	0	**0**
		SEP	Marsabit	0	0	**0**	0	**0**
			Wajir	0	24	**0**	0.1	**0**
			Mandera	0	0	**1**	0	**0**
		NOV	Marsabit	0	135	**2,602**	0.8	**14.7**
			Meru	4.1	1,234	**2,275**	7.0	**12.9**
			Tsavo	9.9	0	**26**	0	**0.1**
			Tana	5	105	**74**	0.6	**0.4**
			Ijara	0	73	**135**	0.4	**0.8**
	2009	FEB	Meru	3.5	555	**363**	3.1	**2.1**
			Wajir	0	1,200	**62**	6.8	**0.4**
			Ijara	0	542	**166**	3.1	**0.9**
		*Totals, Post-Epizootic:*		*5%*	*4,037*	***5,789***	***–***	***–***

Both pre- and post-epizootic sample locations were in areas that had been exposed to risk of RVFV transmission during the epizootic period, as estimated by the risk model. Sample locations from during the epizootic were in areas not flagged as at-risk during the pre- and post-epizootic periods. Thus, despite the lack of contiguous longitudinal spatial/temporal serum samples, the appraised space as a whole may provide insight into the alignment of predictions of RVF activity and observed dynamics of seroprevalence.

## Discussion

The current study was conducted to compare spatial and temporal patterns of available RVF seropositivity data in wild ungulates and camels to zones and timing of potential elevated RVFV transmission predicted by a RVF risk mapping model before, during, and after the 2006–2007 RVF epizootic and epidemic in Kenya. The availability of this unique set of wild ungulate serology data that fortuitously coincided with the location and timing of a major RVF epizootic and epidemic permits two independent ways to look at the outbreak, one through environmentally- and case-based modeling, and one through a landscape-level survey of the indications of infection in wild ungulates. Considering the serology as a single group, even given the limitations of the data (we do not know the animal’s age, exact location, or its full home range), we observe that in inter-epizootic periods, RVF seroprevalence in the wild ungulates sampled is low; during epizootics it is higher. In addition, the study is limited to seroprevalence based on inhibition ELISA results that can vary from the more definitive virus neutralization (VN) assay that requires high biosecurity to perform. Ideally, VN assays would be performed to confirm ELISA-positive samples but unfortunately, the sera collection was damaged due to equipment failure before arrangements could be made. This preliminary appraisal, however, suggests that during RVF outbreaks in domestic animals and humans, we observe that some wild ungulate species also show signs of having been infected. At the landscape level, wild ungulate serology results were generally associated with the predicted patterns of RVFV transmission. Outbreaks of RVF in human populations are generally preceded by epizootics in livestock and it was previously demonstrated that human and livestock RVF cases clustered during this 2006–2007 outbreak in Kenya [28]. Identification of wildlife hosts that may be involved in the epidemiology of RVF, during both inter-epizootic and epizootic periods, is important to not only furthering our understanding of the ecology of this disease, but also in potentially informing and improving the climate-based RVF risk model.

Ungulate samples collected from 2000–2006 had a low seroprevalence, corresponding to the inter-epizootic period when predicted risks were low. Interestingly, in addition to waterbuck and buffaloes, domesticated camels were among the seropositive samples during this timeframe. Camels were heavily impacted in the 2010 RVF outbreak in a non-endemic area of Mauritania [51] and could be among livestock species that could function as particularly sensitive indicators of RVF activity, perhaps because of their longevity and specific range of movement. During the 2000–2006 pre-epizootic period, 7.1% (2/28) of domestic camel samples tested positive, which increased to 57.1% (16/28) testing positive during the 2007 epizootic sampling period (Fig. 2). As the predicted zones of RVF risk in Kenya increased from October 2006, until March 2007, the proportion of positive serum samples similarly increased.

In the years following the 2006–2007 RVF outbreak, predicted risk remained low, as did the seroprevalence of RVFV antibodies in wild ungulate sera collected in 2008–2009. However, the sharp reduction in seroprevalence observed during the post-epizootic period should be interpreted with caution, as it may be an artifact of sampling. On the one hand, some degree of herd immunity would be expected to persist following widespread exposure to the virus; however, the age structure across the samples was not recorded, and these later samples could have been biased towards younger animals born in the months following the 2006–2007 epizootic. Furthermore, the intensity of sampling efforts at each location and with each species varied between 2007 epizootic and 2008–2009 post-epizootic periods. Indeed, the samples available for this study were very limited and collected for reasons not directly related to the study. These factors may help explain the marked decrease in RVF seroprevalence observed during 2008/2009 in some locations. However, our findings do suggest a general alignment between predictions of increasing risk and wild ungulate and camel RVF seroprevalence. For example, results indicate increases in exposure to RVFV inferred from the total population of samples collected in 2007 (31.8%, n = 299), compared to exposure inferred from the total population of samples collected in 2006 (4.3%, n = 94). After the outbreak, there was a reduction in inferred RVFV exposure in the total population of samples from 2008–2009 (5%, n = 358).

During the 2007 epizootic period, we found a large proportion (≥25%) of African buffalo, common eland, giraffe, gerenuk, warthog, impala, and waterbuck sampled at single locations to be seropositive, although sample size was low for some species. A previous serosurvey in Kenya demonstrated that African buffalo, lesser kudu, Thomson’s gazelle, impala, black rhino, and waterbuck sampled during this same outbreak had a relatively high prevalence (≥15%) of neutralizing antibodies against RVFV [42]. Interestingly, that study also suggested that giraffe and warthogs may not be permissive to RVFV replication or that they are not the preferred host of competent vectors based on the low number of seropositive samples. However, the 76.2% (32/42) and 28.6% (2/7) ELISA-positive warthog and giraffe samples, respectively, observed during our study suggest otherwise and indicates how little we know regarding the potential wild ungulate host range.

For each of these four species (African buffalo, common eland, giraffe, and waterbuck) sampled before, during, and after the epizootic, the proportion of positive ELISA results was greater in 2007 samples than in samples from any other year. For instance, seroprevalence in buffalo was zero (0/61) in 2000–2004 samples, slightly increased to 4.8% (1/21) in 2006, and then increased to 21.5% (35/163) in 2007 but was as high as 60% (15/25) in Maasai Mara, followed by a decrease back to 10% or less in 2008 samples. Similar patterns are seen in RVF seroprevalence rates in giraffe and common eland in the years during and adjacent to the epizootic period – yet only the buffalo samples can be compared across years at a single location. Less dramatic shifts in seroprevalence were observed in waterbuck, a grassland-woodland species that generally remains within a few miles of water sources: 16.7% (2/12), 30% (3/10), and 15.4% (2/13) for pre-epizootic, epizootic, and post-epizootic periods, respectively.

Although variable, an increase in RVF seroprevalence rates between pre-epizootic and epizootic periods was observed in camels and in wild ungulate species, including African buffalo, common eland, giraffe, and waterbuck. Although not included in the pre-epizootic sampling period, impala, gerenuk, and warthog all displayed high seropositivity in 2007 samples, and all but impala have samples from the post-epizootic period that demonstrate lower RVF seroprevalence. The above-mentioned species may all represent potential indicators of RVFV activity that future serological studies may include. Gazelle showed some signs of exposure (9.1%, 1/11) to RVFV in Amboseli in late 2006 when RVF risk was beginning to emerge throughout Kenya, but large gazelle samples taken during the peak of the epizootic from epicenters of risk curiously showed no signs of exposure (0%, 0/27). However, this finding should be interpreted with caution. In another study, 87.5% (7/8) of Thomson’s gazelle sampled from Kenya during the 1999–2005 inter-epizootic period tested positive for neutralizing antibodies to RVFV [42].

It is noteworthy that three grassland ungulate species that frequently occupy woodland-grassland edge habitat and do not range far from water sources were all found to be seropositive during the study, these include African buffalo, waterbuck, and impala. Only two impala were sampled, but both tested positive during the 2007 epizootic. Some proportion of serum samples collected from African buffalo (n = 437) and waterbuck (n = 41) during each of the three sampling periods (pre-epizootic, epizootic, and post-epizootic) were positive, suggesting exposure during inter-epizootic periods. These species may be good candidates to include in future longitudinal surveys of wild mammals in ecological zones where RVF may display a more enzootic pattern. However, so little is known regarding natural enzootic and epizootic cycles of RVF in wild mammals, that broader surveys encompassing species from a variety of mammalian taxonomic orders are indicated.

Although the overall percentages of positive serology provide encouraging evidence of linkage between serology and periods of predicted RVFV transmission risk, there are limitations to the conclusions we can make from this opportunistic data set. Limitations include 1) the relatively limited species diversity in the serum samples analyzed, 2) temporal discontinuity of the species sampled, and 3) discontinuity of the geographic locations sampled over time. The first limiting condition is that the majority (78%, 655/840) of samples analyzed were from three species: African buffalo, warthog, and giraffe, with African buffalo comprising 52% (437/840) of the dataset. Seven other wild ungulate species and domestic camels accounted for the remaining 22% (185/840) of serum samples. Another limiting factor was the temporal discontinuity of species sampled (Fig. 2). Of the 11 species sampled across the entire study period, only 4 species, buffalo, eland, giraffe, and waterbuck, have samples from before, during, and after the epizootic. The third limiting condition to conclusions of risk-serology linkage is the non-continuity of locations sampled (Fig. 3). Laikipia and Maasai are the only locations with pre-epizootic samples that may be compared to samples taken during the epizootic, and at both locations buffalo is the only species with samples from both periods and no samples in post-epizootic years for any species. Tsavo East has samples from before and after the epizootic, but not during the epizootic; however, buffalo were sampled in these periods and show 10% positive serology following the epizootic compared to 0% from the pre-epizootic period. No location was sampled in all three periods, but of the 5 locations sampled in 2007 during the epizootic, Isiolo, Maasai Mara, and Lake Naivasha, each showed around 60% seroprevalence across all samples. Data are not available from the epizootic for the majority of locations in this study, yet high RVF seroprevalence indicates that these locations should be among the first to be examined in future, rigorous longitudinal sero-surveys.

Recognizing these limitations, the current study retrospectively supports not only mid-2006 predictions of the 2006–2007 RVF outbreak as published by Anyamba et al. [52], but also furthers our understanding of the apparent epidemiological sequence of RVFV transmission on which the RVF predictive model is based. Global climate anomaly monitoring processed through the RVF predictive model triggered alerts anticipating substantial RVFV transmission from as early as November 2006 at least through January 2007 [52]. In the underlying model of RVFV epidemiology in the RVF predictive system, the initial appearance and transmission of RVFV originates in transovarially RVFV-infected *Aedes* species mosquitoes emerging and surviving in large numbers following periods of anomalously heavy and sustained rainfall and vegetation development [26]. These mosquitoes rapidly begin infecting the primary amplifying hosts, domestic cattle, sheep, and goats. These reservoirs of RVFV replication provide virus for further incidental transmission to humans and other animals via secondary RVFV mosquito vectors, mainly *Culex* species. Although not conclusive, our findings suggest that the appearance of RVF-positive sera in the sampled wild ungulate species lag behind the predicted initiation of the RVF outbreak. As discussed earlier, serosurveys of some wild ungulates in November 2006 were more indicative of inter-epizootic levels of RVF-positive samples. Under the RVFV epidemiological model described in previous studies where the RVF risk assessment model was developed, RVF-positive sera in wildlife are not expected to appear until human cases are observed. Human RVF cases were not observed until December 2006 [31], [32], and in a pattern suggestive of this epidemiological model, the January 2007 serosamples of wild animals described in the present study showed greater presence of RVF than any 2006 samples, though the spatial-temporal distributions of wild ungulate samples were limited as detailed above. Future longitudinal surveys involving both livestock and wild ungulate species can build upon this and related studies and will enable us to better understand the sequence of transmission events before and during an epizootic.

Despite the limitations of this study, we can conclude that the comparison of sample locations to the density of map pixels flagged for RVFV transmission risk (Table 2) did align with the overall observation that seroprevalence rates among the sampled wild ungulate species rises with increasing risk of RVF modeled across the landscape. In addition, this retrospective survey of RVF-positive sera in wild ungulates and camel does not challenge the RVF transmission model. For instance, patterns of change in the presence of RVF-positive sera over time suggest not only that RVFV transmission activity actually took place during a predicted outbreak, but that the timing of transmission to wild ungulates may have been concordant with the RVFV epidemiological model of secondary transmission. However, given the study limitations outlined previously, further research is needed to fully understand the system. Therefore, longitudinal, systematic serological and virological surveillance of wild mammalian species is recommended to establish baseline inter-epizootic antibody levels in wildlife, and thus better track changes in wildlife antibody prevalence with dynamics of modeled RVFV transmission risk levels throughout enzootic regions. Ideally, parallel long-term sero-surveillance studies should also be done in domestic livestock for comparison to changing levels of RVFV transmission risk. Together, these can be used to improve the RVFV transmission risk mapping system by testing and calibrating the underlying epidemiological model.

Sero-surveillance programs should be carried out across a range of environments throughout enzootic regions, including areas inside and outside of the modeled potential epizootic area mask, thus targeting different wildlife species that occupy certain ecological niches. With time, as we begin to better understand the potential role of certain wildlife species in the ecology of RVF, surveillance strategies and sampling efforts can be refined. Future sero-surveillance should include GPS locations of animals at the time of sample and all sampled individuals should be permanently marked. Demographic data should be recorded to better characterize the age structure across the samples which will limit how much of a population sample can be rigorously compared among years. Representative individuals from target species should also be tracked with GPS transmitters to get better information on the movement of herds or individuals as RVFV transmission risk conditions dynamically change over time.

## Conclusions

The combination of climate-based spatial and temporal predictions of RVF risk combined with targeted wildlife and domestic livestock serological and virological surveillance could contribute substantially to understanding RVF epidemiology. The current study retrospectively supports not only mid-2006 predictions of the 2006–2007 RVF outbreak as published by Anyamba et al. [52], but also the epidemiological sequence of RVFV transmission on which the RVF predictive model is based. Future calibration of the RVF transmission risk model will benefit from structured serosurveys of both domestic and wild ungulates throughout the RVF endemic region.
